# Gender- and age-related differences of statin use on incident dementia in patients with rheumatoid arthritis: a Nationwide population-based cohort study

**DOI:** 10.1186/s12944-021-01465-1

**Published:** 2021-04-20

**Authors:** Tsung-Kun Lin, Jing-Yang Huang, Lung-Fa Pan, Gwo-Ping Jong

**Affiliations:** 1 Department of Pharmacy, Taoyuan Armed Forces General Hospital, Taoyuan, Taiwan, Republic of China; 2 School of Pharmacy, National Defense Medical Center, Taipei, Taiwan, Republic of China; 3 Department of Medical Research, Chung Shan Medical University Hospital, Taichung, Taiwan, Republic of China; 4 Institute of Medicine, Chung Shan Medical University, Taichung, Taiwan, Republic of China; 5 Graduate Institute of Radiological Science, Central Taiwan University of Science and Technology, Takun, Taichung, Taiwan, Republic of China; 6 Department of Cardiology, Taichung Armed Forces General Hospital, Taichung, Taiwan, Republic of China; 7 Department of Internal Medicine, Chung Shan Medical University Hospital and Chung Shan Medical University, Taichung, Taiwan, Republic of China

**Keywords:** Gender difference, Dementia, Statin, Rheumatoid arthritis, Population, Cohort study

## Abstract

**Background:**

Some observational studies have found a significant association between the use of statin and a reduced risk of dementia. However, the results of these studies are unclear in patients with rheumatoid arthritis (RA). This study is to determine the association between the use of statins and the incidence of dementia according to sex and age-related differences in patients with RA.

**Methods:**

We conducted a nationwide retrospective cohort study using the Taiwan Health Insurance Review and Assessment Service database (2003–2016). The primary outcome assessed was the risk of dementia by estimating hazard ratios (HRs) and 95% confidence intervals (CIs). Multiple Cox regression was used to estimate the adjusted hazard ratio of new-onset dementia. Subgroup analysis was also conducted.

**Results:**

Among the 264,036 eligible patients with RA aged > 40 years, statin users were compared with non-statin users by propensity score matching at a ratio of 1:1 (25,764 in each group). However, no association was found between the use of statins and the risk of new-onset dementia (NOD) in patients with RA (HR: 1.01; 95% CI: 0.97–1.06). The subgroup analysis identified the use of statin as having a protective effect against developing NOD in male and older patients.

**Conclusion:**

No association was observed between the use of a statin and the risk of NOD in patients with RA, including patients of both genders and aged 40–60 years, but these parameters were affected by gender and age. The decreased risk of NOD in patients with RA was greater among older male patients. Use of a statin in older male (> 60 years) patients with RA may be needed in clinical practice to prevent dementia.

**Supplementary Information:**

The online version contains supplementary material available at 10.1186/s12944-021-01465-1.

## Background

Rheumatoid arthritis (RA) is a common autoimmune disorder that is characterized by systemic inflammatory polyarthritis. It affects approximately 1% of the global population [1, 2]. Previous studies have suggested an increased risk of cerebrovascular and neurodegenerative diseases among patients with RA [3, 4]. Furthermore, an early inflammation and cardiovascular diseases in patients with RA were associated with an increased risk of dementia [5, 6].

There were more than 50 million people living with dementia in 2016 worldwide, and this number is expected to increase with an increase in life expectancy [7]. It is one of the most common causes of disability among this group [8]. Many studies have demonstrated that RA is associated with an increased risk of cognitive decline and dementia [9–11]. Patients with RA and concurrent dementia may put a greater burden on their families and the health care system. Thus, minimizing the impact of incident dementia on patients with RA and their families is a crucial health-related challenge for health care personnel.

It has been suggested that hyperlipidemia is associated with an increased risk of dementia [12]. Statins are effective agents against hyperlipidemia and are widely used in the treatment of cardiovascular diseases [13, 14]. Statin therapy may also reduce the risk of dementia by reducing b-amyloid and serum apolipoprotein levels and exhibiting antithrombotic and anti-inflammatory effects [15]. Some observational studies have found a significant association between the use of statin and a reduced risk of dementia [16, 17]; however, two clinical trials had failed to show its beneficial effects on cognitive function [18, 19]. In fact, these studies presented inconsistent results. Additionally, these previous studies had not focused on patients with RA with respect to sex and age differences, as these patients have a higher risk of dementia. Therefore, the objective of this study is to evaluate the risk of dementia associated with the prescription of statins according to sex and age differences in a nationwide cohort study of patients with RA in Taiwan.

## Methods

### Study design

This is a retrospective cohort study. We used insurance claims data provided by the Taiwanese Bureau of National Health Insurance (BNHI) from January 2003 to December 2016. The BNHI stores complete follow-up information on major interventions and medications as well as admission, outpatient clinic, and emergency department visit records of the Taiwanese population. Statin users were defined as those patients who received statin prescriptions for more than 6 months during the study period and the respective index date was set as the initial statin use day individually. In contrast, non-stain users were designated as those patients who did not receive a statin prescription throughout the whole study period.

### Study population

**Fig. 1:**
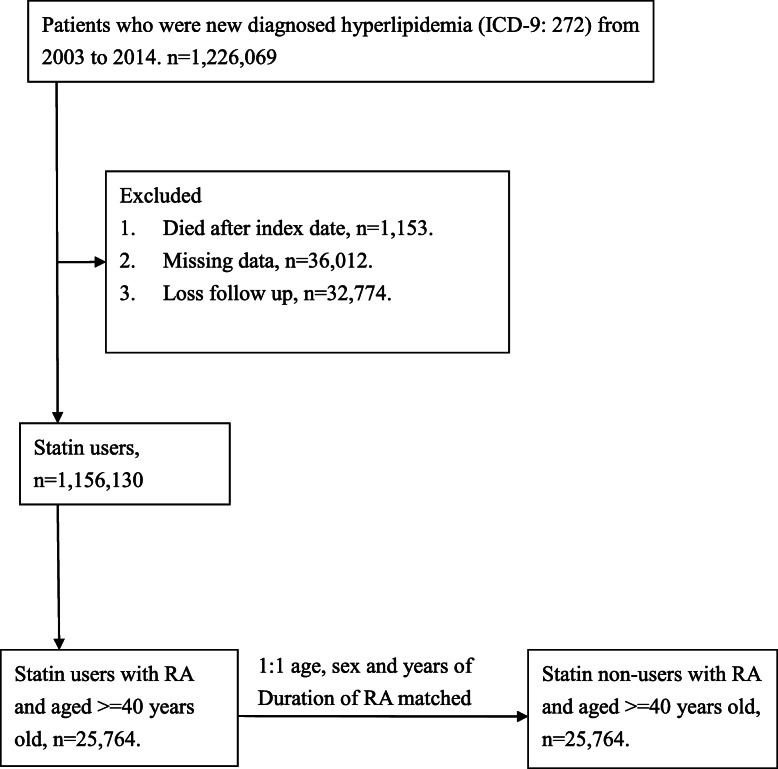
Patient flow chart

The study population comprised patients with RA (International Classification of Diseases, Ninth Revision, Clinical Modification [ICD-9-CM] code: 714.0) who were admitted to the hospital or outpatients who were newly diagnosed with hyperlipidemia (ICD-9-CM code272.X) and were statin users between 2003 and 2016. At least one of the following enrollment criteria had to be met for inclusion in this study: (1) two or more outpatient visits within a six-month period or (2) all statin prescriptions were continuously administered to the patients for more than 6 months within a 14-year follow-up period. Comorbidities related to dementia were as follows: coronary heart disease (ICD-9-CM code 410–415), hypertension (ICD-9-CM code 401–405), diabetes mellitus (ICD-9-CM code 250), and cerebrovascular attack (ICD-9-CM code 430–438). Patients who fulfilled any of the following criteria were excluded from the study: (1) had a prior history of dementia before January 1, 2003 and (2) patients aged less than 40 years. Given the differences in baseline characteristics and dementia risk between the statin users and non-statin users, we applied age, sex, RA duration, and propensity score matching at a ratio of 1:1 for patients with RA with and without statin use. Finally, the study group comprised 25,764 participants with RA who were statin users, and the control group included 25,764 randomly selected participants with RA who were non-statin users (Fig. 1).

### Main outcome measures

The primary endpoint was the development of dementia, which was defined by the time a code of dementia (ICD-9-CM codes 290) first appeared in the inpatient or outpatient claim records.

### Statistical analysis

Initially, the propensity score matching (PSMATCH) was conducted between two groups by using the Statistical Analysis System (SAS) software. The PSMATCH Procedure was used to perform the greedy nearest neighbor matching. First, the logistic regression was performed to estimate the logit probability of exposure to statin for each individual. The details of predictors including RA duration, sex, age, comorbidities, and concurrent medication taken by the patients are listed in Table 1. The algorithm used was the greedy nearest neighbor matching with the caliper of 0.01.

**Table 1:** Baseline characteristics in patients with Rheumatoid Arthritis

	Statin non-user	Statin user	p	ASD
N	25,764	25,764		
Age group			1.0000	0.00000
40–49	3095 (12.01%)	3095 (12.01%)		
50–59	9485 (36.81%)	9485 (36.81%)		
60–69	7469 (28.99%)	7469 (28.99%)		
70–79	4462 (17.32%)	4462 (17.32%)		
> =80	1253 (4.86%)	1253 (4.86%)		
Sex			1.0000	0.00000
Male	7298 (28.33%)	7298 (28.33%)		
Female	18,466 (71.67%)	18,466 (71.67%)		
Co-morbidities				
Ischemic heart disease	6668 (25.88%)	8563 (33.24%)	<.0001	0.16184
Hypertension	13,454 (52.22%)	17,055 (66.20%)	<.0001	0.28735
Ischemic stroke	3298 (12.80%)	4531 (17.59%)	<.0001	0.13367
Diabetes mellitus	7250 (28.14%)	10,236 (39.73%)	<.0001	0.24653
Peptic ulcer	12,575 (48.81%)	12,110 (47.00%)	<.0001	−0.03624
Co-medication				
Aspirin	3035 (11.78%)	6587 (25.57%)	<.0001	0.35953
Beta- blockers	5439 (21.11%)	8076 (31.35%)	<.0001	0.23440
CCBs	6820 (26.47%)	9939 (38.58%)	<.0001	0.26060
ACEI	2035 (7.90%)	3607 (14.00%)	<.0001	0.19625
ARBs	4060 (15.76%)	7321 (28.42%)	<.0001	0.30876
Duration of RA (year)			1.0000	0.00000
< 1	5402 (20.97%)	5402 (20.97%)		
1–3	8018 (31.12%)	8018 (31.12%)		
> 3	12,344 (47.91%)	12,344 (47.91%)		

*ASD* absolutely standardized difference, *CCBs* calicum channel blockers, *ACEI* angiotensin converting enzyme inhibitors, *ARBs* angiotensin receptor blockers

Differences in demographic data and clinical characteristics between statin users and non-statin users were examined using t-test for continuous variables, whereas chi-square tests were used for categorical variables. The dementia-free survival rates in the two groups were calculated using the Kaplan–Meier method and the log-rank test. The Cox proportional hazard regression model was used to compare the developmental risk of dementia (time-to-dementia) between the statin users and non-statin users, while controlling for selected covariates. After the unadjusted model was determined, further adjustments for age, gender, concurrent medication, and comorbidities were included. The hazard ratios (HRs) with 95% confidence intervals (CIs) of incident dementia by the statin users have been reported. *P* values < 0.05 were considered to be statistically significant for this study. All the statistical calculations were performed using statistical analysis software, version 9.3 (SAS Institute, Inc., Cary, NC, USA).

## Results

### Baseline information

Between January 2003 and December 2014, we identified 25,764 patients diagnosed with RA, aged > 40 years, and who were statin users. After 1:1 age, sex, the duration of RA, index date, and propensity score matching, 25,764 non-statin users were used for analyzing the relationship between the use of statin and incident dementia. The baseline characteristics of all patients with RA between the statin user group and non-statin user group are presented in Table 1. The absolute standardized differences between the two groups in all the variables were < 0.1 (10%), and the differences between matched pairs were statistically negligible.

### The relative risk of incident dementia

**Fig. 2:**
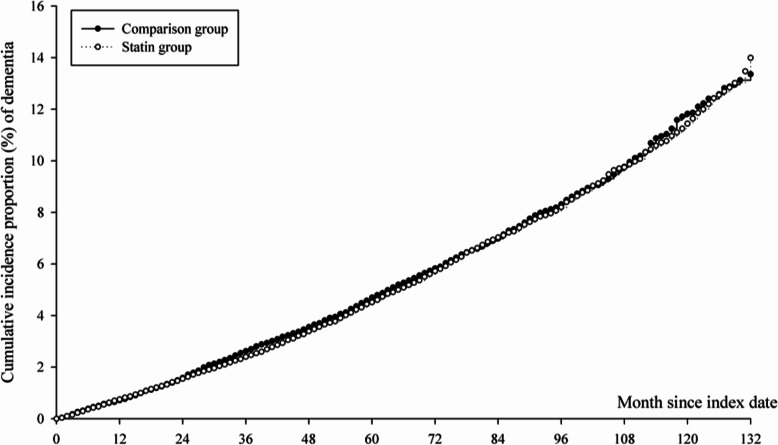
Kaplan–Meier analysis of incidence probability of dementia in patients with rheumatoid arthritis, log rank *p* = 0.6577. Median of follow up time: Non-statin = 61 months, Statin user = 62

Among the 25,764 eligible patients with RA who were statin users, 1562 (6.1%) statin users and 1549 non-statin users (6.0%) developed dementia between 2003 and 2016. During a mean follow-up of 5.8 (SD, 3.6) years, a total 1549 and 1562 incident dementias/1000 person-years among statin users and nonusers (Table 2). There was no significantly difference of the incidence rate of dementia in the statin user group as compared to the non-statin user group (HR: 1.01; 95% CI: 0.97–1.06) (Table 2). The result of the Kaplan–Meier survival analysis also indicated that the risk of developing NODs showed no significantly difference between statin users and non-statin users (*p* = 0.6577) (Fig. 2).

**Table 2:** The incidence rate of dementia in patients with rheumatoid arthritis

	Statin non-user	Statin user
N	25,764	25,764
Follow up person-months	1,689,560	1,707,272
Event of Dementia	1549	1562
Crude hazard ratio (95% CI)	Reference	1.01 (0.97–1.06)

### The relative risk of incident dementia in subgroup analysis

After adjusting sex, age, comorbidities, and concurrent medication, the age subgroup analysis showed a decreasing risk of incident dementia in statin users as compared to non-statin users (adjusted HR: 0.93; 95% CI: 0.86–1.00, *p* = 0.046) in patients with RA aged > 60 years (Table 3). Similarly, male statin users revealed a significantly lower development risk of dementia (adjusted HR: 0.88; 95%CI: 0.81–0.95, *p* = 0.0015) than female patients with RA aged > 60 years. However, the comparison of the duration of RA > 3 or 1–3 years with the duration of RA < 1 year showed no significant association with the developmental risk of dementia in both patients aged > 60 years (adjusted HR: 0.95 and 0.93; 95% CI: 0.86–1.04 and 0.85–1.02) and patients aged 40–60 years (adjusted HR: 1.18 and 1.35; 95% CI: 0.80–1.74 and 0.97–1.88) (Table 3).

**Table 3:** aHRs of dementia in patients with Rheumatoid Arthritis aged 40–60, and > 60 years old

	Aged 40–60 years old			Aged > 60 years old		
	aHR	95% C.I.	p	aHR	95% C.I.	p
Statin use						
Non-user	Reference			Reference		
User	0.94	0.71–1.25	0.6892	0.93	0.86–1.00	0.046
Duration of RA (year)						
< 1	Reference			Reference		
1–3	1.35	0.97–1.88	0.0726	0.93	0.85–1.02	0.1113
> = 3	1.18	0.80–1.74	0.4113	0.95	0.86–1.04	0.2676
Sex						
Female	Reference			Reference		
Male	0.75	0.53–1.06	0.1050	0.88	0.81–0.95	0.0015

aHRs: Adjusted for age, sex, concurrent medication, and comorbidities

## Discussion

This study showed no association between the use of statin and the risk of NOD in patients with RA, but these parameters were influenced by gender and age differences. The decreased risk of dementia in patients with RA was greater in male and older (age > 60 years) patients. This study also demonstrated that RA duration showed no association of dementia incidence between patients who were using statins and patients who were not using statins.

The mechanism underlying the association between statin use and a decreased dementia risk in older male patients with RA may be complex [10, 20]. Several studies showed that systemic inflammation was involved in the pathogenesis of RA and dementia [3, 20, 21]. Additionally, these studies had suggested that an increased systemic inflammation is associated with an increased risk of dementia. Statin treatment is associated with a reduction in serum cytokine levels and C-reactive protein levels that might lower the risk of dementia in patients with RA. Another data show that serum cholesterol levels are linked with the deposition of β-amyloid and pathology of dementia [22, 23]. Statins may reduce the production of b-amyloid by the inhibition of cholesterol biosynthesis to decrease the production of amyloids and it could affect the onset and progression of dementia. However, the decreased risk of dementia in patients with RA was observed only in male patients in this study. Previous observational studies have also found results similar to our study [24, 25]. Zissimopoulos et al. evaluated 399,979 patients and the use of statin was associated with a reduced risk of Alzheimer’s disease in white women and men, Hispanic women and men, and black women. Their study showed that only statin use was not associated with a reduced risk of Alzheimer’s disease among black patients [24]. Finally, they concluded that factors such as race and sex showed an association between the use of statins and the incidence of dementia. Furthermore, it is explained that the clinical response to statins to reduce cholesterol and lipoproteins is variable and related to genetic heterogeneity [26].

Risk factors for dementia include old age and vascular atherosclerosis [27]. Old age causes vascular atherosclerosis, atrophy of the cerebral cortex, and hippocampus, which is associated with memory and spatial impairment. In our study, patients aged > 60 years exhibited a significant protective effect against NODs, whereas patients aged < 60 years did not show any significant association with a risk of developing NODs. This result demonstrates that age difference plays a major role in lowering the risk of NOD by statins among patients with RA. Further comprehensive basic and clinical research is warranted to elucidate the mechanisms underlying this association.

In addition to age, there was a gender-related difference of incident dementia in patients with RA between the statin and non-statin users in our study. Similarly, previous studies have demonstrated gender-related differences in statin treatment across a variety of study populations [24, 28, 29]. These gender-related differences in statin use can be attributed to the following reasons: females were less likely to report having been offered statin therapy, more likely to decline statin therapy when offered, and more likely to discontinue statin therapy after starting [30, 31].

### Strengths and limitations

The strengths of our study are its population-based nature and a large sample size. This study was tested using propensity score matching to control for potential confounders, which established the feasibility of this hypothesis feasible. This study is the first one to provide a comprehensive evaluation on the association between the use of statin and dementia in patients with RA. Moreover, it also found a statistically significant decrease in the risk of NOD among older male patients who are statin users, along with an overall 14% decreased risk as compared to non-statin users. Nonetheless, there were some limitations in this study. Thus, the results should be interpreted with caution. First, our study lacks laboratory data such as blood cholesterol levels, low-density lipoprotein cholesterol levels, renal function, and liver function. Although we used the propensity score matching method to balance a wide range of dementia risk factors between statin users and non-statin users, we cannot exclude the effects of unmeasured confounders. Therefore, there were still other unmeasured confounding factors that may have biased our results. Second, we ascertained that the exposure to statin in the cohort is real and supported by the claims data, which include medication prescription. However, treatment adherence was not available from these secondary data. The protective effect of statins in the development of dementia in patients with RA may be underestimated. Third, we did not document the specific statin types used by the patients as they differ in their pharmacological properties; therefore, we could not evaluate the correlations between the specific statin types used and incident dementia in patients with RA. Finally, this study was based on the Taiwan NHI program and claims data sets, which may be of limited applicability to other countries; therefore, it is unclear that how those findings can be generalized to patient in different areas of the world.

## Conclusion

There is no association between the use of statin and the risk of NOD in patients with RA; however, these parameters are influenced by gender and age. The decreased risk of NOD in patients with RA was greater among male and older patients. We suggested that statin should be used routinely in older male (> 60 years) patients with RA for the prevention of dementia in clinical practice.

## Supplementary Information

**Additional file 1.**

**Additional file 2.**

## Data Availability

The data set analyzed in this study can be reasonably obtained from the corresponding author.

## Abbreviations

CI: Confidence interval
HR: Hazard ratio
ICD-9 CM: International Classification of Diseases, Ninth Revision Clinical Modification
NOD: New-onset dementia
RA: Rheumatoid arthritis
