# Supplementary material for: Gender- and age-related differences of statin use on incident dementia in patients with rheumatoid arthritis: a Nationwide population-based cohort study
Source: Lipids Health Dis. 2021 Apr 20;20:37. doi: 10.1186/s12944-021-01465-1 (PMC8058964; doi:10.1186/s12944-021-01465-1)
Supplement: Supplementary file 1 — Additional file 1. [file 12944_2021_1465_MOESM1_ESM.pdf]

Invoice

付款人姓名: 40201, 臺中市南區建國北路一段110號 中山醫學大學附設醫院

40201, Taiwan, 台中市, 臺中市南區建國北路一段110號
中山醫學大學附設醫院
Contact Person: 國屏 鍾

日期: 2021年2月13日

Invoice #:	IT0252538-21
Invoice Amount	NT$ 1,583
Payment Due Date	2021年2月14日

Details	單價	總計 (TWD)
Inquiry No.: INQ-015907321<br>訂單號碼： GWPJGT-55_On-Hold<br>Service: 標準編修服務 (單次校對)<br>Count: 1,439 字<br>Priority: 經濟件 (Economy)<br>Deadline:2021年2月16日 22:30 臺灣時間<br><br>statin use and dementia risk-M.docx	NT$ 1.1 per unit	1,583
	Premium:	
	Discount:	
	**總計**	1,583

Crimson Interactive Inc.
160 Greentree Dr., Suite 101,
Dover, DE USA 19904
Phone: +1 877 712 2177
Email: payments@enago.com

筆記：

Advertisement

修改發票資訊

若須修改發票抬頭、開立日期等細節，登入會員系統便可迅速修改，還可隨時下載發票。

现在登录: www.enago.tw/mypage

my Editors

自組您的編修團隊！

如果您滿意之前的文稿編修，您可以上系統為編修師按讚，日後將優先為您安排該名編修師。

請登入會員系統**MyPage**使用

詳情請見: www.enago.tw/myeditors
