# Supplementary material for: Gender- and age-related differences of statin use on incident dementia in patients with rheumatoid arthritis: a Nationwide population-based cohort study
Source: Lipids Health Dis. 2021 Apr 20;20:37. doi: 10.1186/s12944-021-01465-1 (PMC8058964; doi:10.1186/s12944-021-01465-1)
Supplement: Supplementary file 2 — Additional file 2. [file 12944_2021_1465_MOESM2_ESM.pdf]

2021年02月17日 10:52AM 1849 個字 • 4 相符 • 1 來源 | 常見問題 |

iThenticate

Gender- and Age-

經由 GWO-PING JONG

包括

包括參

相符處分析

5	**Crossref** 26 個字<br>Chih-Feng Chang, Yi-Sheng Liou, Tsung-Kun Lin, Stacey Ma, Yu-Ru Hu, Hung-Yi Chen, Gwo-Ping Jong. "High exposure to.	1%

符合 1 中的 4

●	**Crossref** 26 個字<br>Chih-Feng Chang, Yi-Sheng Liou, Tsung-Kun Lin, Stacey M a, Yu-Ru Hu, Hung-Yi Chen, Gwo-Ping Jong. "High exposure.	1%

不含來源

純文字報告
